# Bioinformatics construction and experimental validation of a cuproptosis-related lncRNA prognostic model in lung adenocarcinoma for immunotherapy response prediction

**DOI:** 10.1038/s41598-023-29684-9

**Published:** 2023-02-11

**Authors:** Linfeng Li, Qidong Cai, Zeyu Wu, Xizhe Li, Wolong Zhou, Liqing Lu, Bin Yi, Ruimin Chang, Heng Zhang, Yuanda Cheng, Chunfang Zhang, Junjie Zhang

**Affiliations:** 1 Department of Thoracic Surgery, Xiangya Hospital, Central South University, Changsha, 410008 Hunan People’s Republic of China; 2 Xiangya Lung Cancer Center, Xiangya Hospital, Central South University, Changsha, 410008 Hunan People’s Republic of China; 3 National Clinical Research Center for Geriatric Disorders, Xiangya Hospital, Central South University, Changsha, 410008 Hunan People’s Republic of China; 4 Hunan Key Laboratory of Molecular Precision Medicine, Xiangya Hospital, Central South University, Changsha, Hunan 410008 People’s Republic of China; 5 Department of Thoracic Surgery, The Second Xiangya Hospital, Central South University, Changsha, 410011 Hunan People’s Republic of China

**Keywords:** Cancer, Immunology, Biomarkers, Oncology

## Abstract

Cuproptosis is a newly form of cell death. Cuproptosis related lncRNA in lung adenocarcinoma (LUAD) has also not been fully elucidated. In the present study, we aimed to construct a prognostic signature based on cuproptosis-related lncRNA in LUAD and investigate its association with immunotherapy response. The RNA-sequencing data, clinical information and simple nucleotide variation of LUAD patients were obtained from TCGA database. The LASSO Cox regression was used to construct a prognostic signature. The CIBERSORT, ESTIMATE and ssGSEA algorithms were applied to assess the association between risk score and TME. TIDE score was applied to reflect the efficiency of immunotherapy response. The influence of overexpression of lncRNA TMPO-AS1 on A549 cell was also assessed by in vitro experiments. The lncRNA prognostic signature included AL606834.1, AL138778.1, AP000302.1, AC007384.1, AL161431.1, TMPO-AS1 and KIAA1671-AS1. Low-risk group exhibited much higher immune score, stromal score and ESTIMATE score, but lower tumor purity compared with high-risk groups. Also, low-risk group was associated with a much higher score of immune cells and immune related function sets, indicating an immune activation state. Low-risk patients had relative higher TIDE score and lower TMB. External validation using IMvigor210 immunotherapy cohort demonstrated that low-risk group had a better prognosis and might more easily benefit from immunotherapy. Overexpression of lncRNA TMPO-AS1 promoted the proliferation, migration and invasion of A549 cell line. The novel cuproptosis-related lncRNA signature could predict the prognosis of LUAD patients, and helped clinicians stratify patients appropriate for immunotherapy and determine individual therapeutic strategies.

## Introduction

Lung cancer is one of the most common malignancies worldwide, with the high incidence and mortality. In 2020, there was an estimated 2,206,771 newly diagnosed cases and 1,796,144 deaths, respectively^1^. Lung adenocarcinoma (LUAD) is the most frequent histological subtype, taking up 40–50% of lung cancer cases. To date, immune checkpoint inhibitors (ICIs), such as PD-1/PD-L1 and CTLA4, have yielded promising results in LUAD^2–4^. However, major clinical response is only achieved in a small subset of patients. Novel molecular feature stratification of LUAD patients that could accurately predict the efficacy of immunotherapy is urgently needed, and this may help clinicians formulate personalized immunotherapy in the clinic.

LncRNA is a kind of RNA with a molecular weight of more than 200 nucleotides^5^. Nowadays, with the development of high-throughput sequencing, researchers discovered a growing body of lncRNAs that can be deemed as prognostic and stratification biomarkers^6–8^. Besides, accumulating evidence suggested that lncRNAs implied the potential of evaluating the immune cell infiltration and predicting the effect of immunotherapy^9,10^. However, studies concerning lncRNAs as LUAD prognostic biomarkers and their roles in immune regulation and immunotherapy are still insufficient. Further study is warranted.

Recently, a new form of programmed cell death (PCD) was discovered by Tsvetkov et al., termed cuproptosis. Cuproptosis was dependent on mitochondrial respiration and tricarboxylic acid (TCA) cycle^11^. In the present study, we aimed to explore the biological significance of cuproptosis-related lncRNAs in LUAD and constructed a prognostic model. We also evaluated the connection between risk score and tumor microenvironment (TME), tumor mutation burden (TMB) and immunotherapy response. These results might provide us new insights in stratifying patients suitable for immunotherapy and improving the prognosis of LUAD patients.

## Methods

### Data collection and processing

The RNA-sequencing data, clinical information and simple nucleotide variation of LUAD patients were retrieved from TCGA database (https://portal.gdc.cancer.gov/, accessed April 8, 2022). Nineteen cuproptosis-related genes (CRG) were mainly collected from previous study, including LIPT1, GLS, NFE2L2, NLRP3, LIAS, ATP7B, ATP7A, SLC31A1, FDX1, LIPT2, DLD, DLAT, PDHA1, PDHB, MTF1, CDKN2A, DBT, GCSH and DLST^11^. Pearson’s correlation analysis was performed to screen cuproptosis-related lncRNAs (*p* < 0.001, *|R*^*2*^*|*> 0.4). The following R packages were used in this section: ggplot2, dplyr and ggalluvial.

### Construction of LncRNAs Prognostic Model

All patients were randomly separated into training or test cohort with a ratio of 1:1. Uni-Cox analysis was applied to screen cuproptosis-related lncRNAs associated with overall survival (OS) in the training cohort, with *p* < 0.01 considered as significant prognostic factors. Then least absolute shrinkage and selection operator (LASSO) regression analysis was used to narrow down the candidate lncRNAs. Multi-Cox regression analysis was used to construct the risk model and evaluate contribution of each lncRNA as prognostic factors in OS of LUAD cohort. The risk model was calculated as the mathematic formula:

$$ {\text{Risk}}\;{\text{score}} = \mathop \sum \limits_{i = 1}^{n} X_{i} \times \beta_{i} $$

where n, X_*i*_, and β_*i*_ represented total number, FPKM value and regression coefficient of lncRNAs, respectively. High-/low- risk group was divided with the median risk score as cutoff value. Subsequently the patients in the test cohort were also divided into high-/low- risk group based on the defined cut-off value. KM analysis was utilized to evaluate the OS difference in different risk groups. Receiver operating characteristic (ROC) curve was applied to evaluate the diagnostic performance of the model. Uni-cox and multi-cox analysis were applied to evaluate the association between clinicopathological factors and OS. The following R packages were used in this section: survival, ggplot2, caret, glmnet, dplyr, ggalluvial, survminer, pheatmap, timeROC, tidyverse, ggExtra, pec and rms.

### The association between risk score and TMB

Waterfall plot analysis was performed to investigate the association between risk score and TMB. Survival analysis was also performed based on TMB and TMB plus risk score. The following R packages were used in this section: maftools, limma, ggpubr, survival and survminer.

### Functional enrichment analysis

Principal component analysis (PCA) and scatter diagrams were performed. The differentially expressed genes (DEGs) between different risk groups were analyzed by GO and KEGG pathways^12,13^, setting the standards of |*logFC*|> 1 and adjusted *p* < 0.05. The following R packages were used in this section: limma, scatterplot3d, ggplot2, circlize, ggpubr, colorspace, stringi, RColorBrewer.

### The association between risk score and TME

The immune, stromal and ESTIMATEs scores, and the tumor purity was calculated to evaluate the association between risk score and TME. The relative abundances of immune cells were calculated to illustrate the relationship between risk score and immune status. Single sample gene set enrichment analysis (ssGSEA) was applied to investigate the expression differences in immune-related functional gene sets in high- and low-risk groups. The following R packages were used in this section: estimate, CiberSort, ggpubr, ggExtra, GSEAbase, limma, corrplot, and ggplot2.

### Prediction of therapy response

Package “PRRophetic” was used to predict the IC_50_ of the drugs of different groups. Several critical genes associated with the ICIs response were investigated between different risk groups. We employed TIDE database to evaluate the individual likelihood of immune escape. The following R packages were also used in this section: ggpubr, limma, pRRophetic and ggplot2.

### Model comparison and validation

The diagnostic performance of risk model was compared with the existing models collected from literature for LUAD prognosis prediction^14–16^. The clinical and treatment data of IMvigor210 cohort was downloaded from http://research-pub.gene.com/IMvigor210CoreBiologies for model validation. The following R packages were used in this section: survival, caret, glmnet, survminer and timeROC.

### Cell culture

A549 cell line was bought from the Chinese Academy of Science Cell Bank (Shanghai, China), and cultured using RPMI‐1640 (Gibco) medium. LncRNA TMPO-AS1 cDNA was incorporated into the pcDNA3.1( +) vector (Invitrogen) for overexpression of TMPO-AS1 in A549 cell line. The Plasmid was verified by DNA sequencing.

### Cell counting Kit-8 (CCK-8) assay

The cell viability of A549 cell was monitored by CCK-8 (Biosharp). We seeded the A549 cell (3*10^3^ /well) in the 96-well plates, and determined OD450 at 0, 24, 48, 72 and 96 h, respectively.

### Transwell assay

Briefly, 1 × 10^3^ A549 cells were suspended and added into the upper chamber, while RPMI-1640 medium was added into the lower chamber. Cells were incubated at 37 °C for 24 h. Nonmigrating cells were then removed. Migrated cells at the lower surface of the membrane were stained with 1% crystal violet for 15 min, and then assessed.

### Would healing assay

Briefly, confluent satellite cells were serum deprived overnight and then sterile tips were used to scratch on the monolayer. Then media 2% FBS was added to cells for 24 h. The cells were photographed immediately after scratch and at the end of the experiment.

### Statistical analysis

Wilcoxon test was employed for co-expression analysis and differential analysis. Chi-square test was used to investigate the correlation between risk score and clinicopathological features. The Spearman correlation analysis was used for risk score and immune score. The Fisher exact test was used for the differential analysis of TIDE scores. All analysis was two-sided, with *p < 0.05 considered significant. The flowchart was mapped using software EdrawMax (Version 10.5.2, https://www.edrawsoft.com/edraw-max/). Statistical analysis and data visualizations were carried out in R software (R version 4.1.0, Index of /src/base/R-4 (r-project.org)). Image processing was performed using Adobe Illustrator (CC 2017).

## Results

### Cuproptosis-related lncRNAs screening in LUAD patients

**Figure 1:**
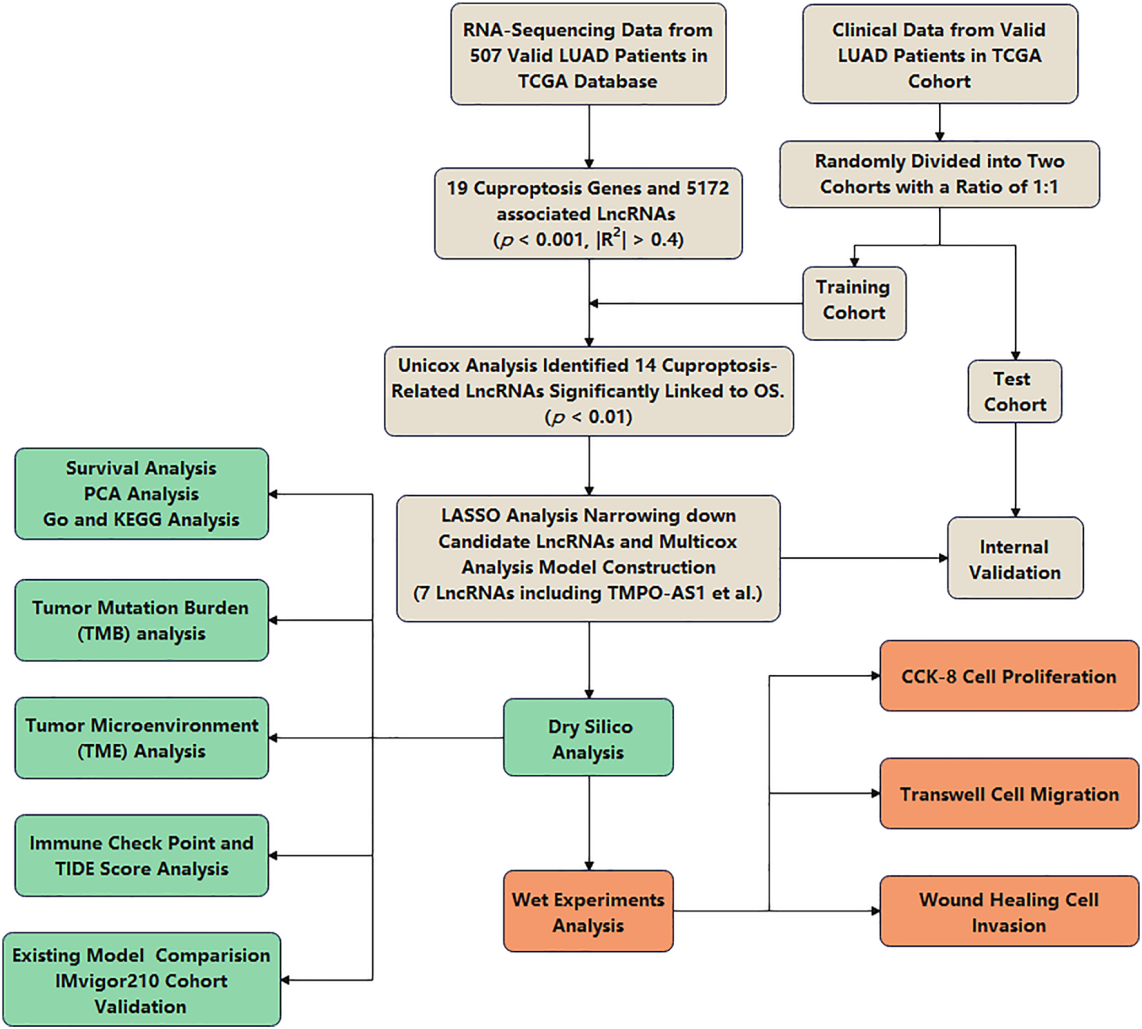
Flowchart of the present study.

**Figure 2:**
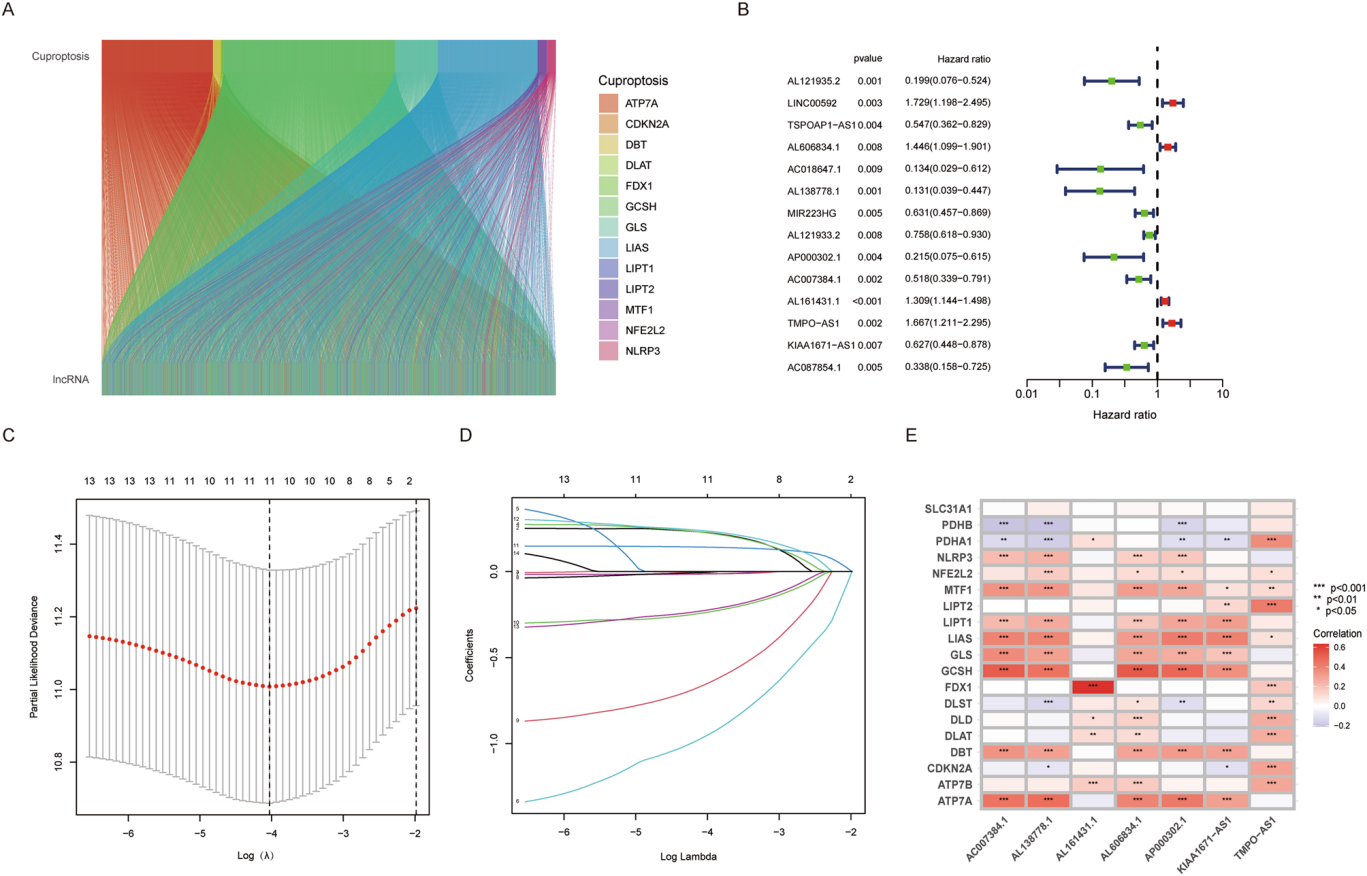
Construction of the Cuproptosis-related LncRNAs Prognostic Signature (**A**) The Sankey diagram shows the connection degree between cuproptosis-related genes and lncRNAs in LUAD patients. (**B**) Forest plots showing the results of the univariate Cox regression analysis between cuproptosis-related lncRNAs and overall survival. (C&D) LASSO regression analysis with a tenfold cross-validation for the prognostic value of the screened lncRNAs. (E) The correlation between lncRNAs screened by multivariate Cox regression and cuproptosis-related genes.

Figure 1 showed the design of the present study. A total of 5172 cuproptosis-related lncRNAs were screened when setting the criteria of* p* < 0.001 and *|R*^*2*^*|*> 0.4. The Sankey diagram of 19 CRGs and lncRNAs was presented in Fig. 2A.

### Construction of risk model using screened lncRNAs

A total of 507 valid clinical data samples of LUAD were collected. The baseline characteristics of training and test cohorts were summarizeds in Table 1. No statistically significant differences were observed between training cohort and test cohort.

**Table 1:** Baseline characteristics of the LUAD patients.

Characteristics	Training cohort	Testing cohort	Total	*P* value
Age				
≤ 65	118	121	239	0.7573
> 65	132	126	258	
Unknown	4	6	10	
Gender				
Female	144	128	272	0.1977
Male	110	125	235	
Stage				
Stage I	133	139	272	0.3311
Stage II	65	55	120	
Stage III	41	40	81	
Stage IV	9	17	26	
NA	6	2	8	
T Category				
T1	94	75	169	0.3865
T2	128	143	271	
T3	22	23	45	
T4	9	10	19	
Tx	1	2	3	
N Category				
N0	162	165	327	0.0837
N1	51	44	95	
N2	35	36	71	
N3	1	1	2	
Nx	5	7	12	
M Category				
M0	176	162	338	0.9102
M1	8	17	25	
Mx	70	74	144	

**Figure 3:**
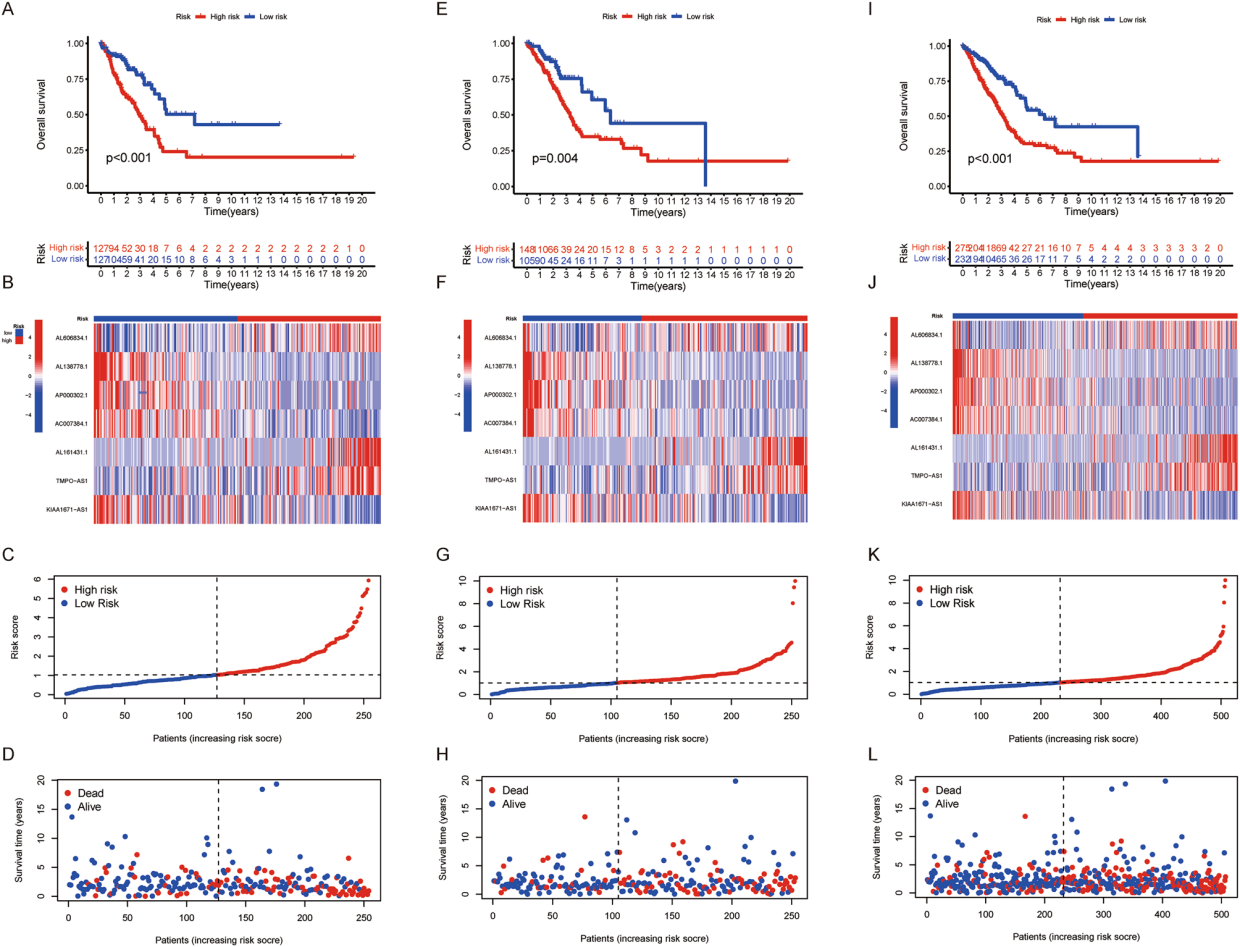
The correlation between the predictive signature and the prognosis of LUAD patients. (**A**–**D**) Training group; (E–H) Test group; (**I**–**L**) Total patients; (**A**&**E**&**I**) Kaplan–Meier analysis of the OS rate of LUAD patients. (**B**&**F**&**J**): Heat map showing the expression profiles of cuproptosis-associated seven-lncRNAs. (C&G&K): The distribution of the risk score among LUAD patients. (**D**&**H**&**l**): The number of dead and alive patients with different risk scores. Blue represents the number of survivors, and red represents the number of deaths.

LUAD: lung adenocarcinoma; NA: not available;

Uni-Cox survival analysis identified 14 cuproptosis-related prognostic lncRNAs in the training cohort (Table 2 and Fig. 2B–D). Subsequently, seven genes cuproptosis-related lncRNA were selected to construct the prognostic score using their regression coefficients, including AL606834.1, AL138778.1, AP000302.1, AC007384.1, AL161431.1, TMPO-AS1 and KIAA1671-AS1 (Table 2). The associations between prognostic lncRNAs and CRGs were shown in Fig. 2E. KM analysis confirmed that low-risk group patients had a better prognosis than those in the high-risk group, with *p* <0.001(Fig. 3A). The heatmap results indicated that the expressions of AL606834.1, AL161431.1 and TMPO − AS1 were upregulated in the high-risk group, while others were downregulated (Fig. 3B). The survival time and living status were displayed in Fig. 3C,D ranked by the distribution of the risk score. Using the same median cutoff value, similar results were observed in the test cohort (Fig. 3E–H) and total cohort (Fig. 3I–L).

**Table 2:** Results of univariate Cox analysis and LASSO analysis.

LncRNA ID	Coefficient	HR	HR.95 L	HR.95H	*p*
AL121935.2	NA	0.199	0.076	0.524	0.001
LINC00592	NA	1.729	1.198	2.495	0.003
TSPOAP1-AS1	NA	0.547	0.362	0.829	0.004
**AL606834.1**	0.282428620063832	1.446	1.099	1.901	0.008
AC018647.1	NA	0.134	0.029	0.612	0.009
**AL138778.1**	− 1.30027751298876	0.131	0.039	0.447	0.001
MIR223HG	NA	0.631	0.457	0.869	0.004
AL121933.2	NA	0.758	0.618	0.930	0.008
**AP000302.1**	− 0.912345907878674	0.215	0.075	0.615	0.004
**AC007384.1**	− 0.325560951666235	0.518	0.339	0.791	0.002
**AL161431.1**	0.164875028131049	1.309	1.144	1.498	<0.001
**TMPO-AS1**	0.337166622358203	1.667	1.211	2.295	0.002
**KIAA1671-AS1**	− 0.271262462650279	0.627	0.448	0.878	0.007
AC087854.1	NA	0.338	0.158	0.725	0.005

Bold biomarker: lncRNAs used for prognostic signature construction; HR. 95 L: hazard ratio 95% low limit; HR. 95 H: hazard ratio 95% high limit; NA: not avaliable.

Moreover, uni- and multi-cox analysis demonstrated that risk score, stage and radiotherapy were independent prognostic factors (Figure S1A&B). The AUC values of the risk model at 1, 2, and 3 years were 0.679, 0.676 and 0.668, respectively (Figure S1C ~ E). The above results suggested that the risk score system might be used as a novel method for stratifying LUAD patients. Then the nomogram was constructed to predict the 1-, 2- and 3-year survival possibility of LUAD patients (Figure S1F&G). The AUC value of nomogram was 0.716, better than the risk model alone (Figure S1H). The slopes of the correction curve were close to 1, indicating a good prediction accuracy of nomogram.

### Functional enrichment analysis of the cuproptosis-related lncRNAs

**Figure 4:**
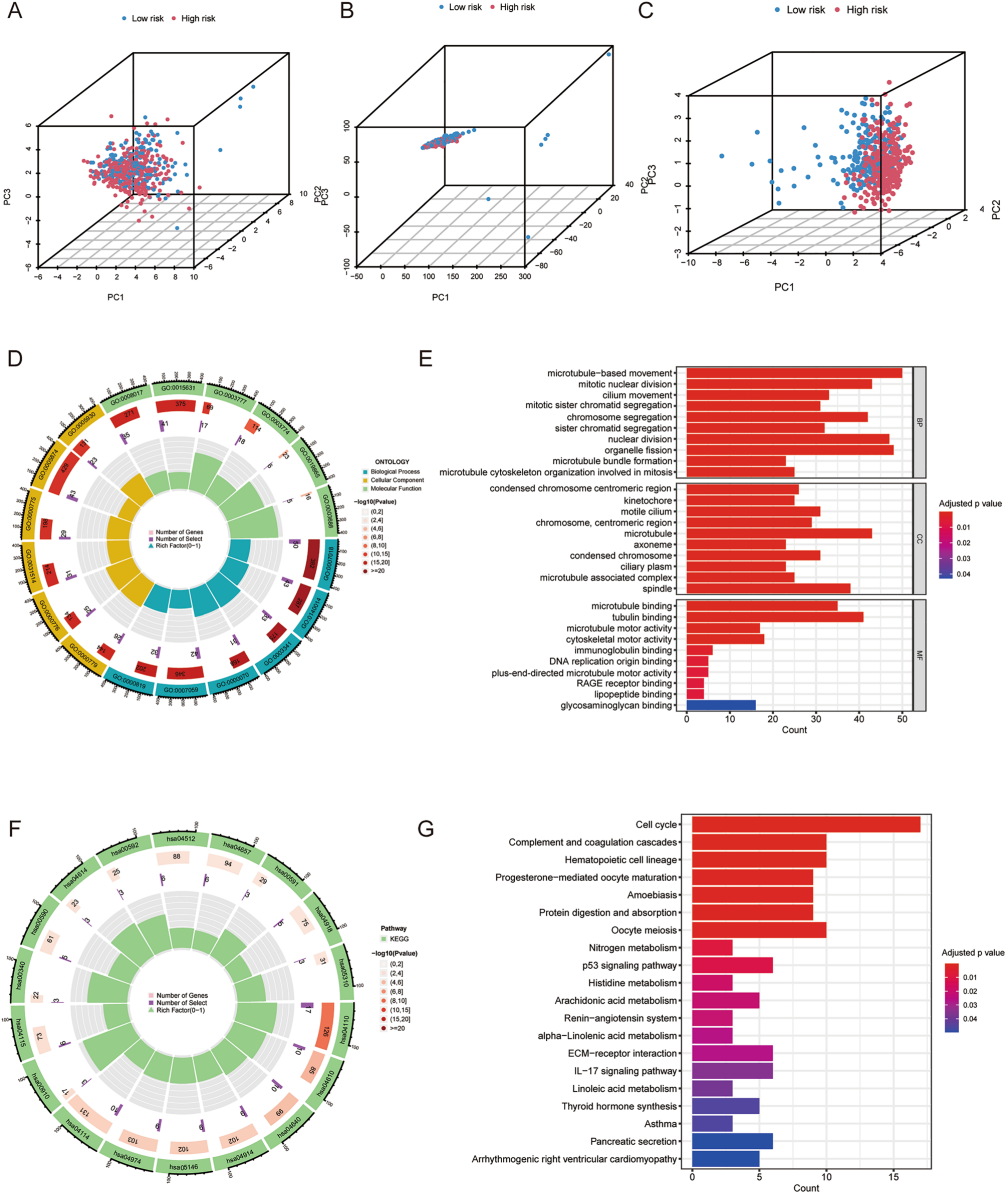
Principal component analyses (PCA) and representative results of functional enrichment analysis. PCA showing the distribution differences between the high- and low-risk groups using cuproptosis-related genes (**A**), cuproptosis-related lncRNAs (**B**) and risk-associated lncRNAs (**C**). (**D**&**E**) GO analysis of biological functions of differentially expressed genes between the high- and low-risk groups. (**F**&**G**) KEGG analysis of biological functions of differentially expressed genes between the high- and low-risk groups.

PCA might reflect that risk score could accurately distinguish different risk groups, while CRGs and cuproptosis-related lncRNAs could not (Fig. 4A–C). GO analysis demonstrated that the biological functions were mainly involved in microtubule binding and microtubule − based movement (Fig. 4D&E). KEGG analysis revealed that DEGs were involved in cell cycle and complement and coagulation cascades (Fig. 4F&G).

### Analysis of TMB in LUAD cohorts

**Figure 5:**
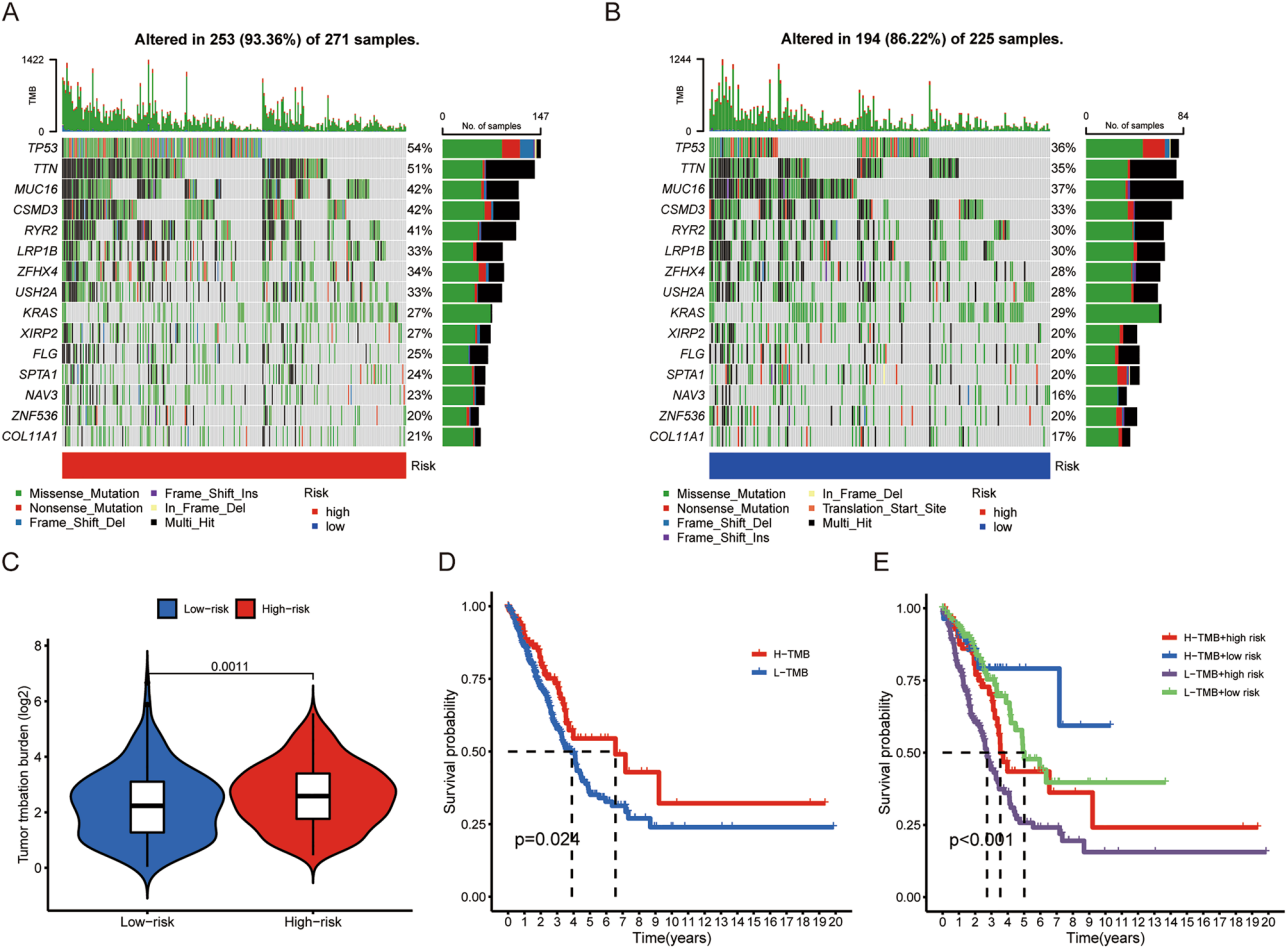
Correlations between the risk score and somatic variants. (**A**, **B**) The mutation rates of top 15 genes in high- and low-risk groups. (**C**) Tumor mutation burden between high- and low-risk groups. (**D**) Correlation between TMB and survival probability in LUAD patients. (**E**) Comprehensive survival analysis based on risk score and TMB.

We then compared the TMB between high- and low-risk groups. High-risk group exhibited a more extensive TMB (Fig. 5A ~ C). The TMB of popular genes in LUAD, such as p53, were 54% and 27% in the high- and low-risk group, respectively (Fig. 5A&B). Previous study indicated that high TMB increased response rates to the immunotherapy and improved outcomes compared with lower TMB^17^. The survival analysis confirmed that high TMB group surely exhibited better OS than the low TMB group (Fig. 5D). Further analysis demonstrated that low-risk patients with H-TMB exhibited the best prognosis among the four subgroups (Fig. 5E). This inspired us that the combination of risk score and TMB might have a more accurate prognostic value for LUAD patients.

### The association between risk score and TME

**Figure 6:**
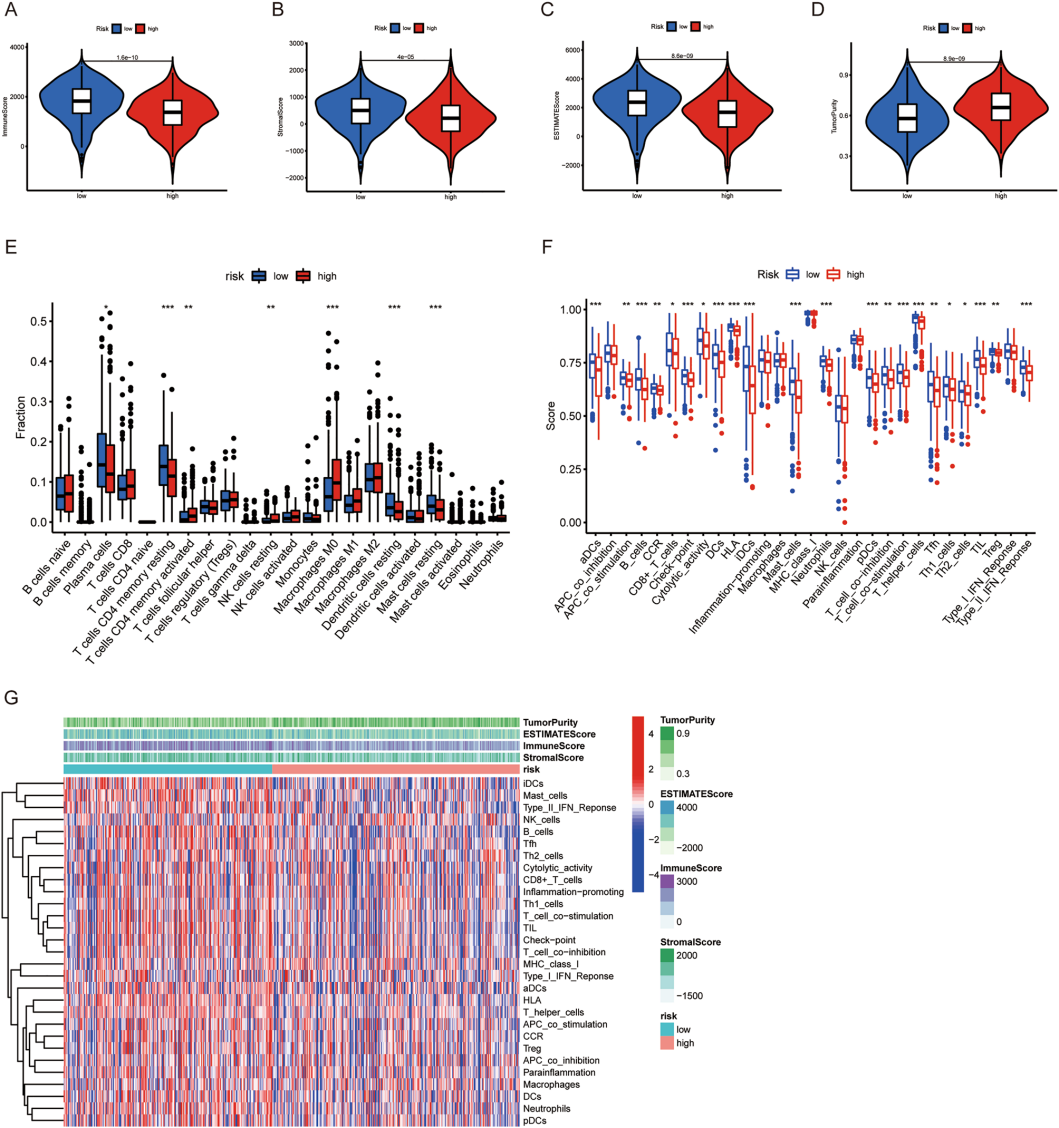
Correlation between the risk score and tumor microenvironment in the LUAD patients. (**A**–**D**) Comparison of immune scores, stromal scores, ESTIMATE scores and tumor purity between the high- and low-risk groups. (**E**) The abundance difference of 22 immune cells in the high- and low-risk groups. (**F**) Enrichment differences in immune function-related gene sets between high- and low-risk groups. (**G**) Heatmap of the TME score and immune gene expression of LUAD patients in different risk groups.

As shown in Fig. 6A–D, low-risk group exhibited much higher immune score, stromal score, ESTIMATE score, but lower tumor purity compared with high-risk groups, with *p*<0.001. Low-risk group was associated with more abundant plasma cells and resting CD4 memory T cells and resting dendritic cells, while high-risk group was accompanied with richer activated CD4 memory T cells and M0 macrophages (Fig. 6E). Besides, low-risk group exhibited a much higher score in nearly all immune cells and immune related function sets compared (Fig. 6F,G).

### Prediction of chemotherapy and targeted therapy response in different risk groups

Six common drugs used in lung cancer exhibited a significant IC50 difference between high- and low-risk groups, including 5-fluorouracil, gemcitabine, mitomycin C, vinorelbine, paclitaxel and alectinib. Interestingly, all 6 drugs showed a much lower IC_50_ values in the high-risk group, indicating a better antitumor efficacy (Figure S2).

### The association between risk score and immune checkpoint genes

**Figure 7:**
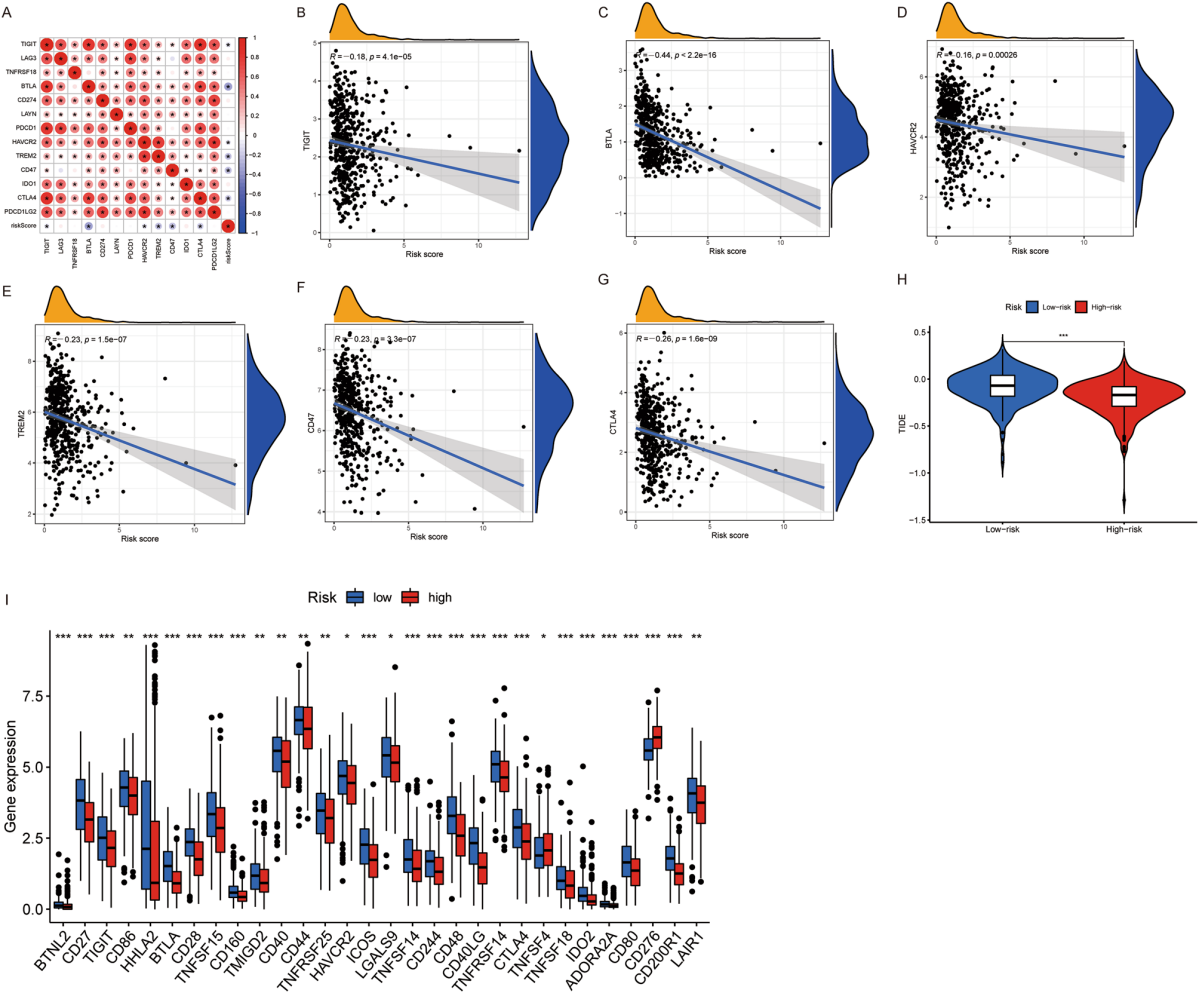
The association between risk score and immune checkpoints. (**A**) The correlation between critical immune checkpoint-related genes and risk score; Red color depicts a positive correlation, while blue color represents a negative correlation. The darker color intensity and larger circle represent a stronger correlation. * indicates *p* < 0.05, the correlation is significant. (**B**–**G**) Scatter plots and straight regression lines demonstrated the correlation between the risk score and significant genes related to immune checkpoints. (**H**) The TIDE score distribution between high- and low-risk groups. (**I**) The expression difference of 47 immune checkpoint-related genes between high- and low-risk groups.

Six genes, including TIGIT, BTLA, HAVCR2, TREM2, CD47 and CTLA4, exhibited a strong negative correlation with risk score, with *p*<0.001 (Fig. 7A–G). The low-risk group patients presented higher TIDE scores compared with high-risk group, indicating a much more potential of immune escape (Fig. 7H). Also, nearly all immune checkpoint-related genes showed a relatively high expression in the low-risk group, but except CD276 (Fig. 7I). Given the high expression of CTLA-4 in the low-risk group, patients might be more sensitive to respond to anti-CTLA4 immunotherapy (Fig. 7B).

### Model comparison and validation

We compared the diagnostic performance of the risk model with the existing models collected from literature for LUAD prognosis prediction^14–16^. The AUC values of risk model in the present study, Zhang’s model, Song’s model and Ren’s model were 0.679, 0.687, 0.668 and 0.674, respectively (Figure S3A). Our model exhibited a comparable and even better diagnostic performance compared with the existing models.

For further validation our risk model in predicting immunotherapy response, we collected the clinical and treatment data from IMvigor210 cohort: http://research-pub.gene.com/IMvigor210CoreBiologies and calculated risk score of each patient. Low-risk group exhibited a better prognosis, with *p* < 0.05 (Figure S3B). We then compared the risk score distribution of each group, including inflamed, excluded and desert. Inflamed subgroup exhibited relatively lowest risk scores, while desert group exhibited highest risk scores, with statically significant differences, which indicated a more abundant immune infiltrations in inflamed subgroup. Also, the immunotherapy response results demonstrated that CR/PR group had lower risk scores compared with SD/PD groups, with *p* < 0.05 (Figure SS3D). Thus, these results might indicate that low-risk group more easily benefited from immunotherapy.

### TMPO-AS1 overexpression promotes the proliferation, migration and invasion of A549 cell line

**Figure 8:**
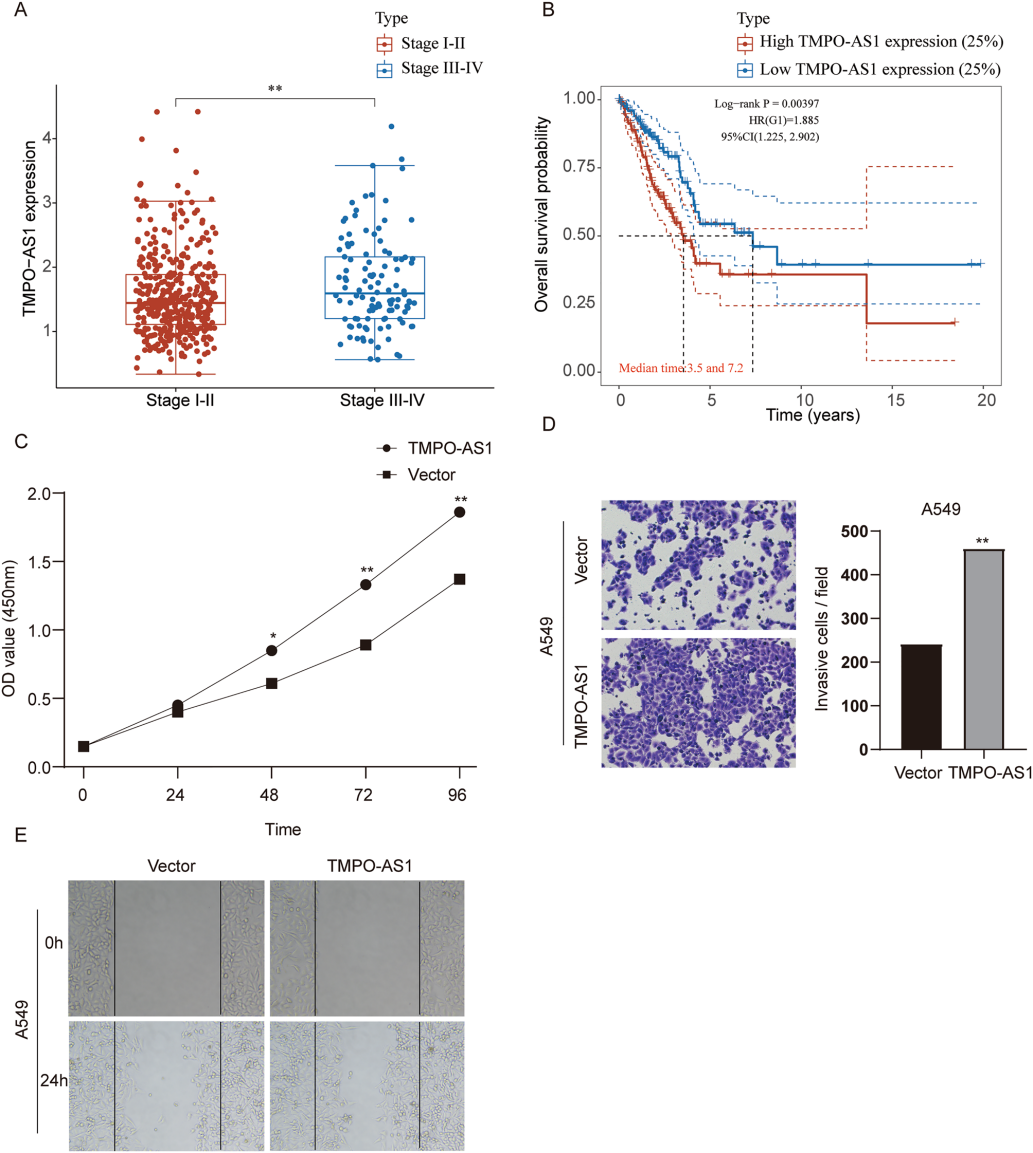
The effect of TMPO-AS1 overexpression on A549 cell proliferation, migration and invasion. (**A**) Relative TMPO-AS1 expression of Stage III-IV LUAD patients compared with Stage I-II LUAD patients analyzed with the TCGA database. (**B**) Association of TMPO-AS1 expression with overall survival of LUAD patients. (**C**) The effect of TMPO-AS1 overexpression on the viability of A549 cell line. (D&E) The effect of TMPO-AS1 overexpression on the migration and invasion of A549 cell line. *p < 0.05, **p < 0.01. Experiments were repeated three times.

The HR value of TMPO-AS1 was the most significant among the 7 lncRNAs used for risk mode construction. Thus, TMPO-AS1 was chosen for further in vitro validation. The expression level of TMPO-AS1 in Stage III-IV LUAD patients was significantly higher than that in Stage I-II LUAD patients from TCGA database (Fig. 8A). Also, high TMPO-AS1 expression patients (top 25%) exhibited a poorer prognosis than low TMPO-AS1 expression patients (Fig. 8B). In vitro experiments further validated that overexpression of TMPO-AS1 promoted the proliferation, migration and invasion of A549 cell line (Fig. 8C–E). In summary, TMPO-AS1 increases the tumorigenicity of A549 cell line.

## Discussion

The risk model consisted of 7 lncRNAs, of which AL606834.1, AL161431.1 and TMPO-AS1 had been previously reported. The high expression of AL606834.1 was associated with poor prognosis and unfavorable immune response in LUAD patients^18^. Down-regulation of AL161431.1 might suppress the proliferation, migration of A549 cells, and induced cell apoptosis^19^. TMPO-AS1 was regarded as an oncogenic lncRNA and reportedly participated in the occurrence of various tumors^20–22^. In vitro studies, high expression of TMPO-AS1 was demonstrated to function as a sponge of ceRNAs and inhibit miRNA expression by targeting and binding with miRNAs. This might lead to the overexpression of downstream genes, and facilitate tumor initiation^22^. Relatively little is known about other 4 lncRNAs. Further studies to investigate the role of these lncRNAs are needed.

By immune infiltration analysis, the abundance of immune cells, including B cells, DCs, mast cells and neutrophils were richer the in low-risk group. The B cells are carriers of humoral immunity, and they can secrete immunoglobulins, regulate T cell response and play an anti-tumor role^23^. Dendritic cells (DCs) are professional antigen presenting cells (APCs) that connect the innate and adaptive branches of the immune system^24^. Mast cells induce the release of angiogenic and lymphangiogenic factors, and thus promote the formation of blood and lymphatic vessels^25^. High neutrophils infiltration was reported to promote the metastasis of LUAD and resistance to chemotherapy^26,27^. Thus, pro- and anti-tumorigenic immune cells co-existed in the LUAD tissue and leaded to a complex TME. In addition to immune cells, a variety of immune system process pathways were highly involved in the low-risk group, such as cytolytic activity. High CYT score was accompanied with cytotoxic T cell markers and good prognosis in pan-cancer TCGA datasets^28^. Our survival analysis results were also consistent with this conclusion.

TMB, TIDE score and TME have been proposed as indicators for prediction of immunotherapy response^29–32^. Considering that high TMB is associated with a greater possibility of displaying tumor neoantigens on HLA molecules^33^, it is rational to hypothesize that high-TMB tumor is more likely to benefit from immunotherapy. However, sometimes neo-antigens recognized by T cell may not originate in a high mutation setting. Large numbers of mutations do not contribute to the development of tumor-specific neoantigens and stimulation of the immune system^34^. Thus, TMB might not always correlate with immunotherapy response^35^. TIDE score is primarily calculated based on limited gene expression biomarkers to model the immunotherapy efficacy comprehensively. But some key biomarkers that can predict T cell infiltration and immunotherapy response, such as β-catenin protein level, were not incorporated into the prediction algorithm^36^. As the authors proposed, more data types and methods supplement are necessary to improve the predictive performance of TIDE score^36^. Another problem is that TIDE score has not been tested in a real-world cohort but only in theory, and its predictive accuracy for immunotherapy response may need further validation.

Compared with TMB and TIDE score, the immune contexture and tumor infiltration lymphocytes (TILs) are the direct reflections of the immune status, and tightly associated with anti-tumor efficiency^37^. The expression of immune check point genes, such as CTLA-4 and PD-L1, have been applied and tested in the clinic to predict the immunotherapy response. In the present study, although low-risk group exhibited a low TMB and high TIDE score, TME analysis indicated a more abundant TILs, and CTLA-4 expression was also higher in the low-risk group. The expression of novel immune check point genes, such as TIGIT, BTLA and CD47, were negatively correlated with risk score, with *p*<0.001. External validation using real-world IMvigor210 immunotherapy cohort demonstrated that low-risk group also had a better prognosis. Thus, we tend to conclude that low-risk group may more easily benefit from immunotherapy even if the indicators’ results were a little conflicting. But this conclusion may need further validation using real-world cohort. Besides, it is possible that risk score, TME, TMB and TIDE score could be applied jointly to achieve a more accurate and higher prediction performance in the future.

The induction of PCD in cancer cells was considered as the most promising anti-tumor strategy. Previously discovered PCD types, such as ferroptosis and pyroptosis, have exhibited great significance in the treatment of LUAD^38,39^. However, the exploitation of copper toxicity in cancer was not successful^11^. An important reason why copper is not effective, is the absence of useful biomarkers for selecting patients appropriate for treatment. Results of elesclomol from phase 3 clinical trials in unselected melanoma patients demonstrated lack of efficacy, but a post hoc analysis confirmed evidence of anti-tumor activity in patients with low-LDH levels^40^. In the present study, the connection between cuproptosis and lncRNA-based model was established, and the model demonstrated great potential for predicting OS and stratifying patients for immunotherapy. Whether the model can be used to predict the copper toxicity in cancer is worth further study.

Recently, with the rapid development of bioinformatics, researchers and clinicians try their efforts to uncover the nature of various carcinomas using TCGA and GEO databases. He et al. constructed a 5-methylcytosine-related risk model to predict the prognosis and immunotherapy response in lung squamous cell carcinoma patients^41^. Wang et al. applied immune-related lncRNA pairs to construct a prognostic signature to reveal the immune landscape of stomach adenocarcinoma^42^. As for LUAD, Liao et al. analyzed the immune cell infiltration in LUAD to predict the effect of immunotherapy^43^, and identified novel prognostic biomarkers of LUAD based on cancer stem cell theory using weighted gene co-expression network analysis^44^. In the present study, we constructed a novel risk model based on cuproptosis-related lncRNAs in LUAD, validated the risk model in external database, and compared the predicting performance with other existing models reported in the literature. The model exhibited a good and comparable performance and might be used as a novel method for predicting LUAD patients’ prognosis in the future.

Several limitations needed to be addressed. Firstly, the research was based on bioinformatics analysis, prospective real-world data to verify the clinical utility of the model would be more convincing. Secondly, wet experiments were superficial, and molecular interactions between lncRNAs and cuproptosis-related genes needed to be further characterized. Thirdly, the relatively small sample size and partial missing data also caused some bias.

## Conclusion

In summary, we constructed a novel risk model based on cuproptosis-related lncRNAs, which could predict the LUAD patients’ prognosis and stratify patients suitable for immunotherapy. The risk model might help clinicians determine individual therapeutic strategies in the future.

## Supplementary Information

Supplementary Information 1.

Supplementary Information 2.

Supplementary Information 3.

Supplementary Information 4.

## Data Availability

RNA-sequencing data and clinical data used were downloaded from the TCGA database.
