# Supplementary material for: Bioinformatics construction and experimental validation of a cuproptosis-related lncRNA prognostic model in lung adenocarcinoma for immunotherapy response prediction
Source: Sci Rep. 2023 Feb 11;13:2455. doi: 10.1038/s41598-023-29684-9 (PMC9922258; doi:10.1038/s41598-023-29684-9)
Supplement: Supplementary file 1 — Supplementary Information 1. [file 41598_2023_29684_MOESM1_ESM.docx]

**Figure S1**: (A&B) Univariate analysis and Multivariate analysis of the lncRNA risk score and clinical features. (C) ROC curve of the risk score and clinicopathological characteristics in predicting the overall survival. (D) ROC curve and AUC of the cuproptosis-related lncRNA model at 1-year, 2-years and 3-years in the LUAD cohort. (E) Calibration curves showed the consistency between predicted and actual survival. (F) The calibration charts showed that the predicted OS results were in good agreement with the actual clinical observations. (G) A combined nomogram for the risk score model and other clinicopathological factors. (H) ROC curve of the nomogram in predicting the overall survival

**Figure S2**: The IC_50_ distribution difference of 6 commonly used drugs between high- and low-risk groups. (A) 5-fluorouracil; (B) Gemcitabine (C) Mitomycin C, (D) Vinorelbine, (E) Paclitaxel and (F) Alectinib.

**Figure S3**: (A) Model comparison; (B) KM analysis of IMvigor210 cohort using our risk model; (C) Risk score distribution of Desert, Excluded and Inflamed subgroup in the IMvigor210 cohort; (D) Risk score distribution of CR/PR and SD/PD subgroup in the IMvigor210 cohort;
