# Supplementary figures and images for: Bioinformatics construction and experimental validation of a cuproptosis-related lncRNA prognostic model in lung adenocarcinoma for immunotherapy response prediction
Source: Sci Rep. 2023 Feb 11;13:2455. doi: 10.1038/s41598-023-29684-9 (PMC9922258; doi:10.1038/s41598-023-29684-9)

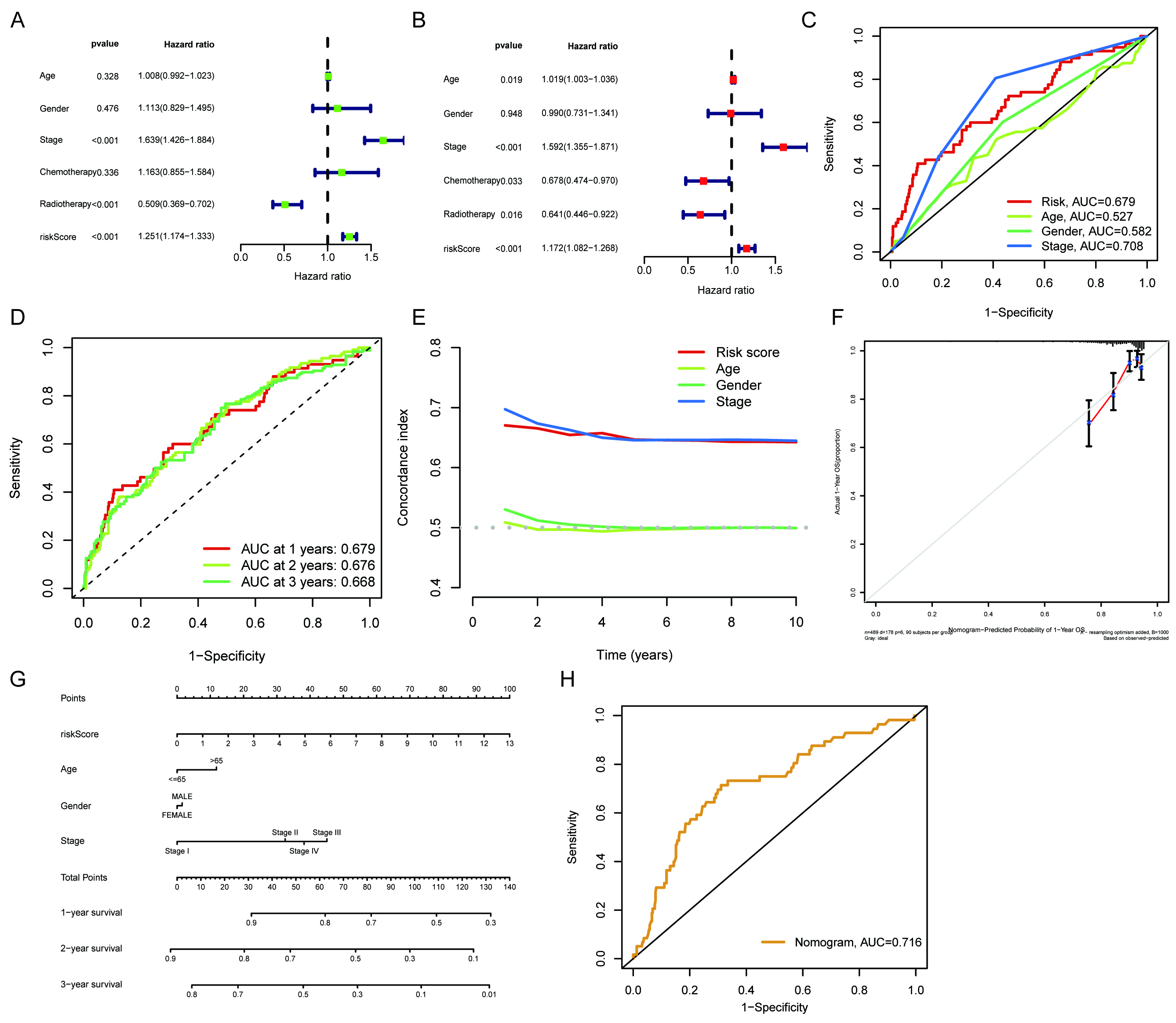

Supplement: Supplementary file 2 — Supplementary Information 2. [file 41598_2023_29684_MOESM2_ESM.tif]

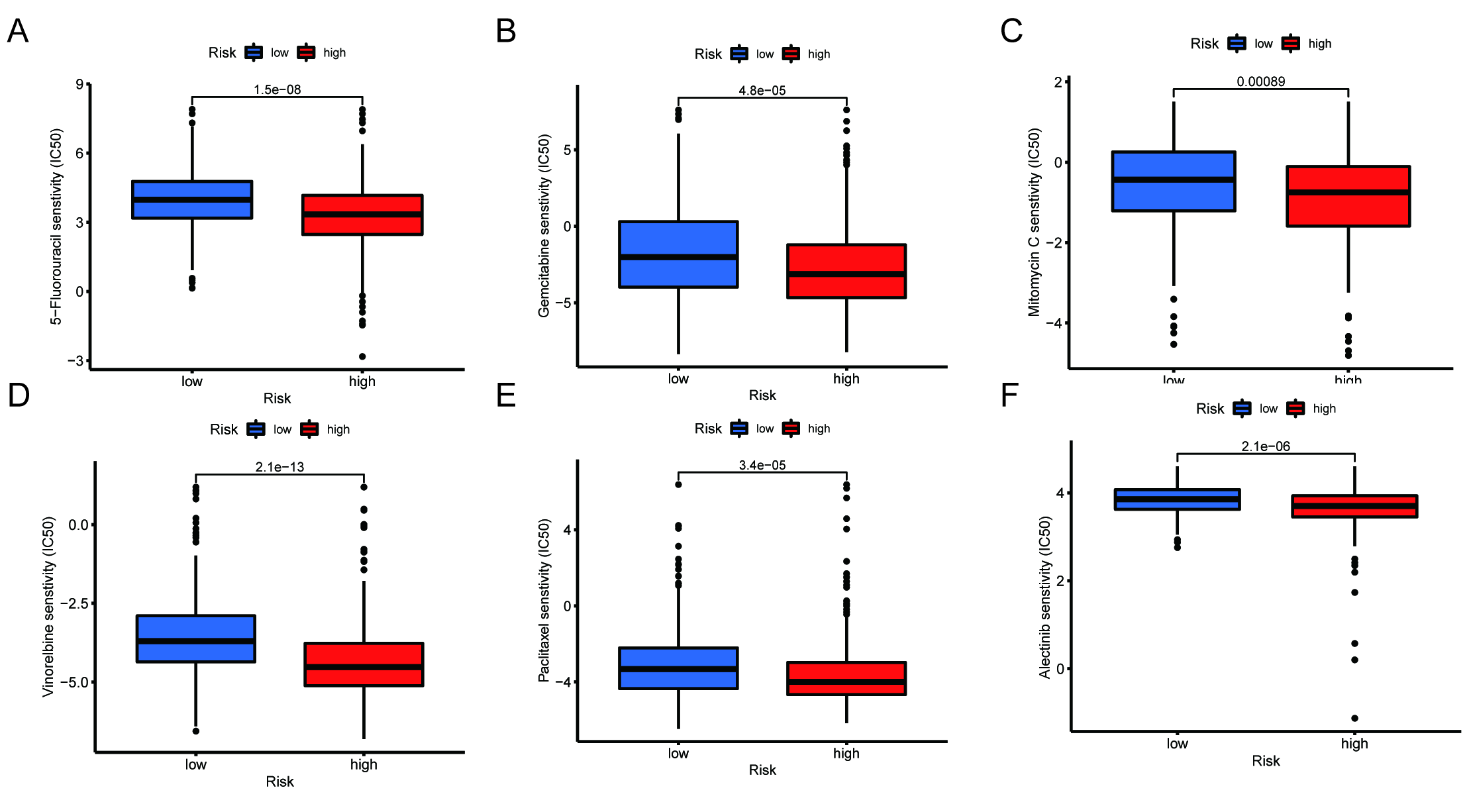

Supplement: Supplementary file 3 — Supplementary Information 3. [file 41598_2023_29684_MOESM3_ESM.tif]

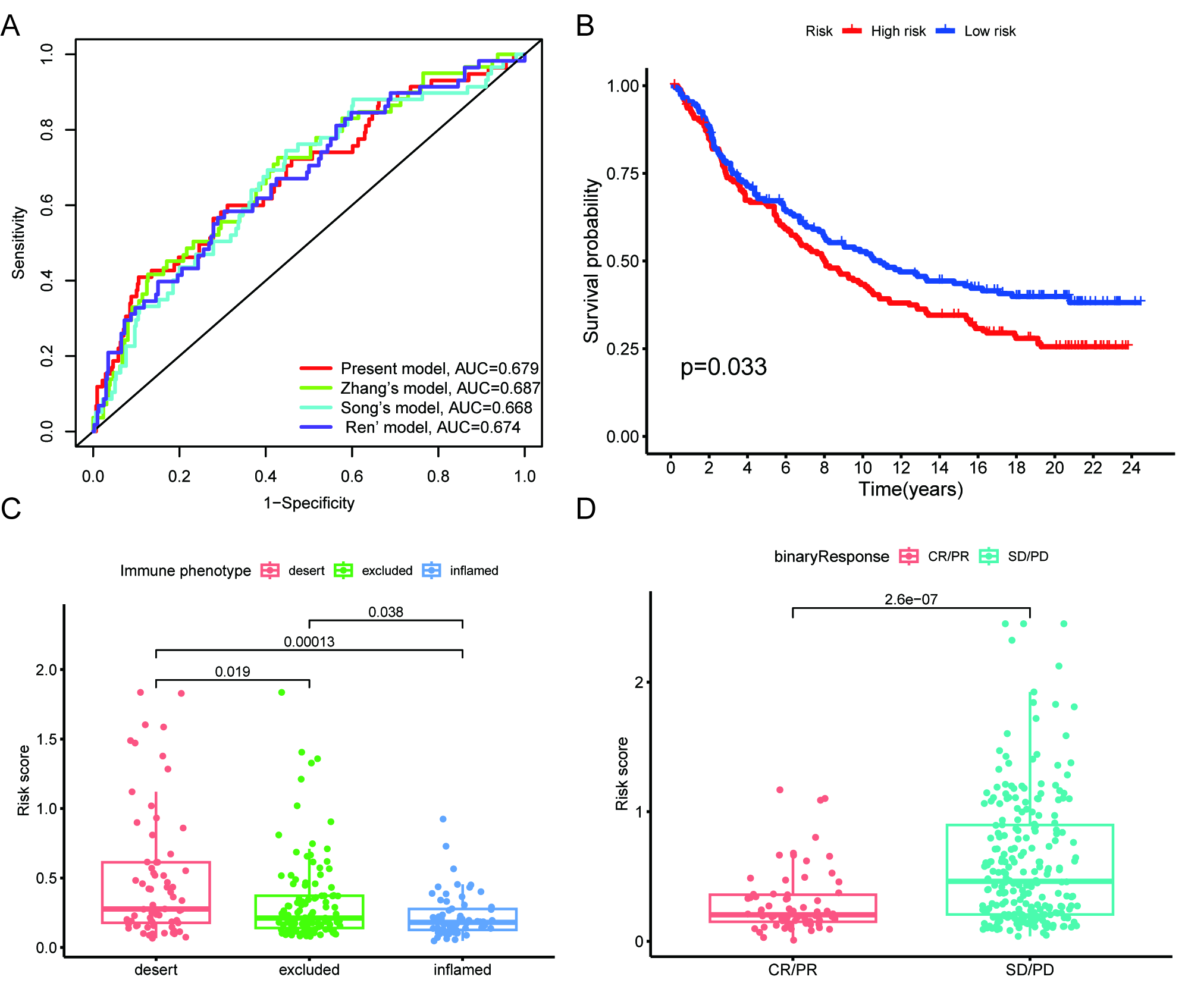

Supplement: Supplementary file 4 — Supplementary Information 4. [file 41598_2023_29684_MOESM4_ESM.tif]
